# Return on investment for implementation of perioperative goal-directed therapy in major surgery: a nationwide database study

**DOI:** 10.1186/cc14266

**Published:** 2015-03-16

**Authors:** F Michard, M Krukas, F Ernst, S Fogel

**Affiliations:** 1 Edwards Lifesciences, Irvine, CA, USA; 2 Premier Inc, Charlotte, NC, USA; 3 Carilion Clinic, Roanoke, VA, USA

## Introduction

Preventable postsurgical complications are increasingly recognized as a major healthcare burden. A recent meta-analysis showed a 17 to 29% decrease in complications after major surgery with perioperative goal-directed therapy (PGDT) [1]. We assessed the financial consequences of postsurgical complications in a large population from 541 US hospitals in order to predict potential savings with PGDT.

## Methods

Data from adults who had any one of 10 major noncardiac surgical procedures between January 2011 and June 2013 were selected from the Premier research database. Twenty-six postsurgical complications were tabulated. Hospital costs, length of stay, and readmission rates were compared in patients with and without complications. Risk ratios reported by Pearse's meta-analysis were used to estimate the expected reduction in postsurgical morbidity with PGDT. Potential cost-savings were calculated from the actual and anticipated morbidity rates using the mean difference in total costs.

## Results

A total of 204,680 patients met the search criteria, and 76,807 patients developed one or more postsurgical complications (morbidity rate 37.5%). In patients with and without complications, hospital costs (including 30 days readmission costs) were $27,607 ± 32.788 and $15.783 ± 12,282 (*P *< 0.0001), median (interquartile range) hospital lengths of stay (first stay) were 7 (4 to 10) days and 4 (3 to 5) days (*P *< 0.0001), and 30-day readmission rates were 17.2% and 11.9% (*P *< 0.0001), respectively. With PGDT, the morbidity rate was anticipated to decrease from 26.6 to 31.1%, yielding gross cost savings of $153 million to 263 million for the study period, $61 million to 105 million per year, or $754 to 1,286 per patient.

## Conclusion

Postsurgical complications occurred in more than one-third of our study population and had a dramatic impact on hospital costs, length of stay, and readmission rates. Potential cost savings with PGDT were $754 to 1,286 per patient. These projections should help hospitals estimate the return on investment for implementation of PGDT.
